# Dosimetric comparison of library of plans and online MRI-guided radiotherapy of cervical cancer in the presence of intrafraction anatomical changes

**DOI:** 10.1186/s13014-019-1322-0

**Published:** 2019-07-12

**Authors:** J. Visser, P. de Boer, K. F. Crama, Z. van Kesteren, C. R. N. Rasch, L. J. A. Stalpers, A. Bel

**Affiliations:** 1 Department of Radiation Oncology, Amsterdam UMC, University of Amsterdam, Meibergdreef 9, Amsterdam, the Netherlands; 2 Present Address: Radiotherapeutisch Instituut Friesland, Borniastraat 36, Leeuwarden, the Netherlands; 3 Present Address: Department of Radiation Oncology, Leiden University Medical Center, University of Leiden, Albinusdreef 2, Leiden, The Netherlands

**Keywords:** Cervical, Intrafraction, MRI-guided, Adaptive

## Abstract

**Background:**

Online magnetic resonance imaging (MRI)-guided radiotherapy of cervical cancer has the potential to further reduce dose to organs at risk (OAR) as compared to a library of plans (LOP) approach. This study presents a dosimetric comparison of an MRI-guided strategy with a LOP strategy taking intrafraction anatomical changes into account.

**Methods:**

The 14 patients included in this study were treated with chemo radiation at our institute and received weekly MRIs after informed consent. The MRI-guided strategy consisted of treatment plans created on the weekly sagittal MRI with 3 mm and 5 mm planning target volume (PTV) margin for clinical target volume (CTV) cervix-uterus (MRI_3mm and MRI_5mm). The plans for the LOP strategy were based on interpolations of CTV cervix-uterus on pretreatment full and empty bladder scans. Dose volume histogram (DVH) parameters were compared for targets and OARs as delineated on the weekly transversal MRI, which was acquired on average 10 min after the sagittal MRI.

**Results:**

For the MRI_5mm strategy D_98%_ of the high-risk CTV was at least 95% for all weekly MRIs of all patients, while for the LOP and MRI_3mm strategy this requirement was not satisfied for at least one weekly MRI for 1 and 3 patients, respectively. The average reduction of the volume of the reference dose (95% of the prescribed dose) as compared to the LOP strategy was 464 cm^3^ for the MRI_3mm strategy, and 422 cm^3^ for the MRI_5mm strategy. The bowel bag constraint V_40Gy_ < 350 cm^3^ was violated for 13 patients for the LOP strategy and for 5 patients for both MRI_3mm and MRI_5mm strategy.

**Conclusions:**

With online MRI-guided radiotherapy of cervical cancer considerable sparing of OARs can be achieved. If a new treatment plan can be generated and delivered within 10 min, an online MRI-guided strategy with a 5 mm PTV margin for CTV cervix-uterus is sufficient to account for intrafraction anatomical changes.

**Trial registration:**

NL44492.018.13.

**Electronic supplementary material:**

The online version of this article (10.1186/s13014-019-1322-0) contains supplementary material, which is available to authorized users.

## Background

Cancer of the uterine cervix affects predominantly relatively young women (30–60 years of age) in the prime of their lives. In western countries, more than 40% of the patients have locally advanced disease for which the first choice of treatment would be chemo radiation, which leads to a 5-year survival rate of 65% [1, 2]. Surviving women have to live with a 87% late toxicity rate, of which 11% is ≥ grade 3 [3, 4]. Not surprisingly, current radiotherapy practice innovation focuses on better local control by better tumour coverage and dose to organs at risk (OAR) reduction [5].

For external beam radiation therapy (EBRT) with volumetric-modulated arc therapy (VMAT), image-guided adaptive radiation therapy with a library of plans (LOP) approach became state-of-the-art care during the last decade, leading to a margin reduction and less dose to OAR [6, 7]. In the past decade, with magnetic resonance imaging (MRI) and adequate brachytherapy target coverage aided by interstitial needles, better local control is achieved while severe toxicity rates are declining [2, 8, 9]. Traditionally, tumour extension in cervical cancer could hardly be visualised on CT and additionally, radiotherapy techniques would not allow highly conformal dose delivery around planning target volumes (PTV) with complex three-dimensional shapes [10]. Nowadays, also for external beam radiation therapy (EBRT) the importance of determining tumour extension on MRI further increases as margins are being reduced with plan-of-the-day adaptive strategies [11].

With reasonable local control, focus of EBRT improvements lies on reducing large margins and consequently aiming to reduce acute and late small bowel toxicity [12]. Probably more small bowel could be spared if treatment plans could be adjusted for tumour shrinkage with MRI-guided radiotherapy with online replanning. This concept seems promising since cervical tumours shrink on average approximately 50% after two-third of the EBRT fractions [13, 14]. Furthermore, margins for geometrical uncertainties would only have to compensate for intrafraction motion, whereas compensation for interfraction motion, which implies rather large margins, would at last be put out of game [15–18].

Multiple studies have compared online MRI-guided radiotherapy with standard conformal radiotherapy. In this study, MRI-guided radiotherapy is compared with the current state-of-the-art treatment, LOP. Furthermore, the dosimetric effect of intrafraction motion is taken into account.

## Methods

### Patients

Patients included in this study were treated for locally advanced cervical cancer at our institute between January 2016 and November 2017. During the inclusion period of this study patients were treated at our institute with chemo radiation which consisted of EBRT followed by a brachytherapy boost with concurrent weekly 40 mg/m^2^ cisplatin. As part of the clinical protocol, patients underwent a computed tomography (CT) scan for delineation and treatment planning (slice thickness 2.5 mm, Light speed RT16, GE Healthcare) with a full bladder and an empty bladder as part of a LOP strategy, which is described elsewhere [19]. One hour before acquisition of the full bladder CT, patients were instructed to drink 1 l of water with contrast fluid to visualize the small bowel. After voiding the bladder the empty bladder CT scan was acquired. One liter of water is more than patients were instructed to drink prior to a treatment fraction, because for the LOP strategy the extremes of the bladder filling were required. This way the target volumes during the treatment were expected to be interpolations between target volumes on full and empty bladder scan. For delineation purposes an MRI in treatment position with an empty bladder was made (Ingenia 3.0 T MR system, Philips) where the scan protocol included anatomical T2-weighted turbo spin echo sequences (TE = 80 ms, TR = 4000 ms) with 0.6 × 0.7 × 3 mm^3^ resolution, acquired in transversal, coronal and sagittal orientation. During the inclusion period the pretreatment MRI was used as empty bladder scan to replace the CT with empty bladder. Because of anatomical changes during the course of the treatment some patients received additional CTs with full and empty bladder and a new LOP was created.

### Weekly MRI

As part of the study protocol, all included patients received weekly MRIs during EBRT. Patients who received less than three weekly MRIs were excluded from the analysis. The acquisition of the extra MRIs was approved by the local medical ethics committee and patients were included after obtaining informed consent.

The anatomy of the patients during the weekly MRIs was supposed to be representative of a treatment fraction. Therefore, the weekly MRI scans were acquired in treatment position using the same scan protocol as used for the pretreatment MRI. Both for the weekly MRIs and the treatment fractions the same drinking instructions were given. Furthermore, if the weekly MRI was acquired prior to the treatment fraction, patients were instructed to void their bladder after the MRI acquisition and drink 500 ml of water. If the weekly MRI was acquired after the treatment fraction, patients were instructed to empty their bladder after the treatment fraction and drink 500 ml of water before the acquisition of the MRI. In case the pretreatment MRI for delineation was not used for the LOP, it was considered to be one of the weekly MRI scans.

### Delineation

For this study the target structures were delineated in Velocity (Velocity 3.2, Varian Medical Systems) by a radiation oncologist resident (PB) according to the delineation guidelines in the EMBRACE II study protocol [20]. If clinical delineations were present, they were evaluated and adapted if necessary. Pathologic lymph nodes were not delineated and not taken into account. The following target structures were delineated: GTV_T, consisting of the cervix tumor; CTV_T_HR, the high-risk clinical target volume (CTV), consisting of GTV_T and the remaining cervix; CTV_T_LR, the low-risk CTV, consisting of CTV_T_HR with a 5 mm margin in the anterior-posterior direction but not extending into rectum and bladder, the uterus, the parametria both sides, and the upper 2 cm of the vagina; CTV_LN_Pelvic, the lymph node regions. The upper boundary for CTV_LN_Pelvic was the aortic bifurcation, as all patients in our study were classified as intermediate risk.

On the pretreatment transversal MRIs the bladder and CTV_T_LR were delineated. All target structures were delineated on all available weekly transversal and sagittal MRIs, except for CTV_LN_Pelvic which was partially outside the field of view (FOV) of available weekly MRIs. A bony anatomy match (Velocity) was used to propagate CTV_LN_Pelvic from the full bladder planning CT scan to the weekly MRI scans. As a consequence, shape changes due to bladder and bowel filling were neglected. Organs at risk (OAR) were delineated by an experienced radiation therapist (MB). The bowel bag was delineated on the empty bladder planning CT scan. For patients who did not receive an empty bladder CT scan, the bowel bag was first delineated on the full bladder planning CT scan and then caudally extended using the fused weekly transversal MRI to obtain the delineation of the empty bladder bowel bag as visible on the weekly transversal MRI. The bladder was not delineated on the weekly sagittal MRIs, because the bladder was partially outside the FOV for most patients. The rectum was delineated on all weekly MRI scans.

### LOP strategy

For this study the full bladder planning CT scan, empty bladder CT or pretreatment transversal MRI scan, including bony anatomy registration between these scans (Velocity), and all delineations were imported in RayStation (RayStation v6.99, RaySearch). The delineations of the low-risk CTV and bladder on full and empty bladder scan were used as input for the contour-based biomechanical deformable registration algorithm in RayStation [21]. Interpolations of the low-risk CTV were obtained using the scripting interface by applying the deformation vector field to the vertices of the low-risk CTV on the full bladder scan and scaling the resulting difference vector. The number of interpolations was the least number of interpolations for which the maximum distance between the interpolations did not exceed 1 cm. For each interpolation and both delineations of the low-risk CTV on full and empty bladder scan an anisotropic internal target volume (ITV) margin was applied: 10 mm in anterior and posterior direction; 5 mm in left, right, inferior, and superior direction, where inferior to the vaginal part of the low-risk CTV the margin was 0 mm. Then encompassing CTVs were created by applying a union with lymph node regions CTV. PTVs were obtained by applying an isotropic expansion of 5 mm, which is equal to the PTV margin used in the EMBRACE II study [20].

For treatment plan optimization purposes OAR delineations were created for each plan in the LOP. The bladder was interpolated using the same deformation vector field and scaling factors as for the low-risk CTV. The delineation of the rectum on the full bladder planning CT scan was used for optimization of each plan in the LOP. Interpolations of the bowel bag were created by subtraction of rectum, interpolated cervix-uterus and bladder from the empty bladder bowel bag.

For the plans in the LOP VMAT dual arc was used, where the energy was 10 MV and the arc length was 357° with one control point every 3° of gantry angle. The clinical beam model for an Elekta Agility linac was used, the dose grid resolution was 3 mm in all directions, and the full bladder planning CT scan was used for dose calculation. The plans in the LOP were created using the Plan Explorer module for automated planning in RayStation where the prescribed dose was 45 Gy in 25 fractions. The wish list with clinical goals is given in Additional file 1: Table S1. Sparing of OARs was not allowed to compromise target coverage (D_98%_ > 95%) and therefore the clinical goal related to PTV coverage was given the highest priority.

The clinical workflow was followed in our study; if a patient received additional CTs and a new LOP was created during the course of the treatment, also for this study a new LOP was created, where the same scans were used as in the clinic.

### MRI-guided strategy

An online MRI-guided strategy was simulated by creating a treatment plan for each of the weekly MRIs. Delineations on the weekly sagittal MRIs were used to create two treatment plans with different PTV margins. The full bladder CT scan was used as planning CT, thereby neglecting the effect of anatomical changes on the dose distribution. Delineations on the weekly sagittal MRIs were propagated to the full bladder CT scan using a bony anatomy match with only translations (Velocity). Remaining rotations reflected the day-to-day setup rotation error. This was achieved by creating a bony anatomy match with both rotations and translations and then removing the rotations using the top of the uterus on the midline of the patient as the rotation point, as this was approximately the center of the total target volume. After importing the delineations in RayStation an encompassing CTV was created by a union of the low-risk CTV and the lymph node regions CTV. Two PTVs, MRI_3mm and MRI_5mm, were created with an isotropic expansion of the low-risk CTV of 3 mm and 5 mm, respectively. This way it was possible to test whether 3 mm or 5 mm PTV margin was sufficient to maintain target coverage in the presence of intrafraction motion of the cervix-uterus. In both cases an isotropic PTV margin of 3 mm was applied to the lymph node regions CTV, which was less than the margin for the LOP strategy, because it was expected that for the LOP strategy a larger margin was required to cope with interfraction setup rotations.

For treatment plan optimization purposes a bowel bag delineation was created by subtraction of the low-risk CTV and rectum from the bowel bag delineation on the empty bladder scan. This particular bowel bag delineation also contained the bladder, which was not delineated in the weekly sagittal MRIs because it was partially outside the FOV. Treatment plans for MRI_3mm and MRI_5mm were designed for the whole treatment of 25 fractions in the same way as for the LOP. The wish list is shown in Additional file 1: Table S2 and was the same wish list as used for the LOP, but without clinical goals for the bladder, which was not delineated.

### Evaluation of planned dose

For each treatment plan the following target dose volume histogram (DVH) parameters were obtained for the planned dose: PTV D_98%_ and D_0.1%_, both expressed as percentage of the prescribed dose. To check target conformity of the planned dose the conformity index (CI) was determined, which was defined as the volume of the reference dose divided by the target volume covered by the reference dose, where 95% of the prescribed dose was used as the reference dose [22]. In R [23], for each parameter the Kruskal-Wallis test was used to test differences between the set of all LOP plans, the set of all MRI_3mm plans, and the set of all MRI_5mm plans, where a difference was considered significant if *p* < 0.05. Since the treatment plans were created with different OAR delineations, no comparison of planned OAR dose was done.

### Estimated fraction dose including intrafraction motion

The weekly MRIs consisted of sagittal, coronal and transversal MRIs, which were acquired in this order. The difference in time between the acquisition of the sagittal and transversal MRIs was used to simulate intrafraction motion. The treatment plans for the different strategies were evaluated using the delineations on the weekly transversal MRIs. This way, the effect of intrafraction motion was simulated for the evaluation of the MRI-guided strategy, since the plans for the MRI-guided strategy were based on delineations on the weekly sagittal MRIs. For each patient the average time difference between the weekly sagittal and transversal MRIs was calculated using the acquisition time.

For the LOP strategy, the effect of the intrafraction motion was simulated by using each weekly sagittal MRI for the selection of the best fitting plan in the LOP, and using the subsequent transversal MRI for the evaluation of the selected plan.

**Fig. 1:**
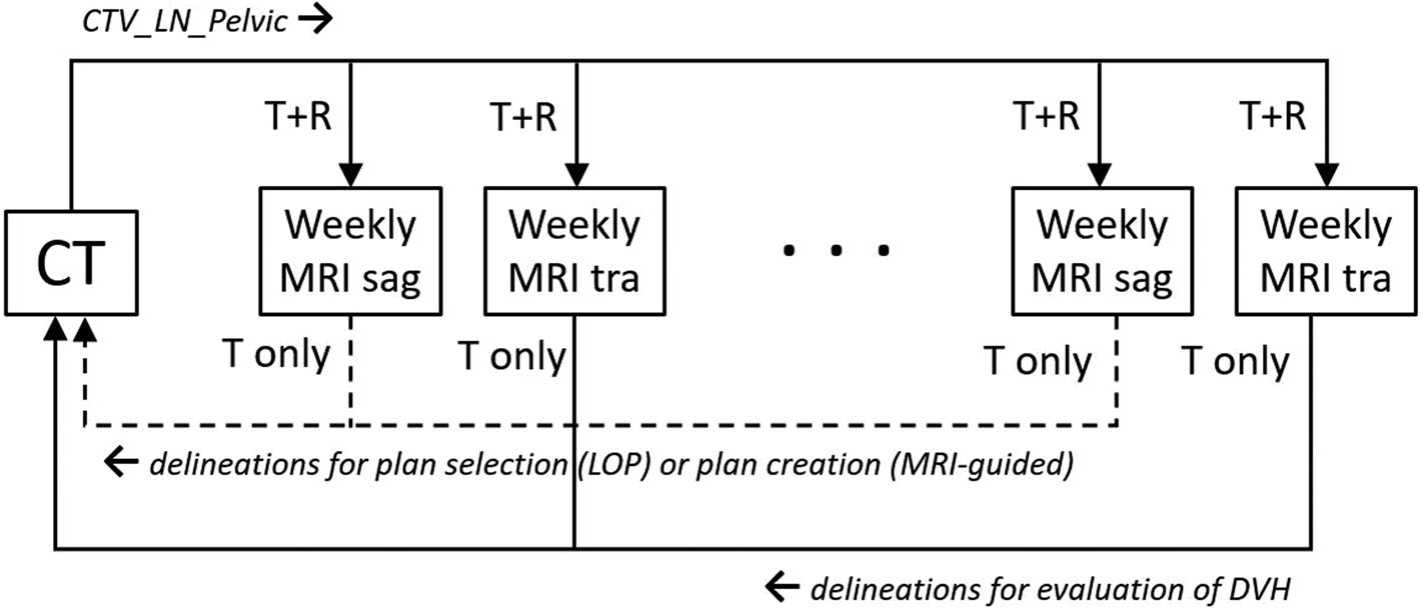
Overview of the propagated delineations between the weekly MRIs and the planning CT. CTV_LN_Pelvic was propagated to the weekly MRIs using a bone match with translation and rotations (T + R). The other targets were delineated both on weekly MRI sag and MRI tra. Delineations on weekly MRI sag were propagated to the planning CT using a bone match with translations only (T only) for the purpose of plan selection (LOP strategy) or plan creation (MRI-guided). Delineations on weekly MRI tra were propagated to the planning CT in the same fashion and used for the dosimetric evaluation

The delineations on the weekly transversal MRIs were propagated to the full bladder planning CT in the same way as the delineations on the weekly sagittal MRIs, that is, using a bony anatomy match without rotations (Velocity), where the top of the uterus was used as the rotation point for removing the rotation from the match. This also mimics the clinical setup protocol for LOP, where the rotation point lies in the center of the total target volume. By doing this, possible setup translations between the sagittal and transversal MRIs were ignored. The overview in Fig. 1 shows which registrations were used for propagation of the delineations between the weekly MRIs and the planning CT.

After importing the delineations in RayStation, for each patient and each weekly MRI the DVHs for the different strategies were calculated using the fractional dose distribution multiplied by 25, the prescribed number of fractions. This enabled straightforward comparison with DVH parameters for the whole treatment.

For the different strategies D_98%_ was determined for the low-risk CTV (CTV_T_LR), the high-risk CTV (CTV_T_HR), and the lymph node regions CTV (CTV_LN_Pelvic), expressed as a percentage of the prescribed dose. For each patient the average volume of the reference dose (95% of prescribed dose), which is a measure for target conformity, was determined for the different strategies (LOP, MRI_3mm, and MRI_5mm). For the LOP strategy the average volume of the reference dose was determined by taking the weighted average volume of the reference dose over all plans in the LOP, where the weight was equal to the number of times a plan was selected. For the MRI-guided strategy the average volume of the reference dose of the plans created for the weekly sagittal MRIs was determined.

DVH parameters were determined for the OARs. For rectum and bladder these were D_mean_(Gy) and V_40Gy_(%). Bowel bag V_40Gy_(cm^3^) and V_30Gy_(cm^3^) were determined, and for each patient the average of these parameters was calculated over the weekly MRIs. It was checked if the average V_40Gy_ and V_30Gy_ satisfied the dose constraints for acute bowel toxicity suggested by Fiorino et al. [24], that is, V_40Gy_ < 350 cm^3^ and V_30Gy_ < 500 cm^3^.

For the bowel bag it was hypothesized that the potential dosimetric benefit of the MRI-guided strategy, as compared to the LOP strategy, increases with increasing bowel bag dose. This was tested by creating a linear regression model in R for the bowel bag V_40Gy_ difference between both the MRI_3mm and MRI_5mm strategy and the LOP strategy, as a function of the bowel bag V_40Gy_ for the LOP strategy. The hypothesis was rejected if the linear coefficient in the model was not significantly different from zero (*p* ≥ 0.05).

For each patient, each weekly MRI, and each OAR DVH parameter, the difference was determined between the value of the DVH parameter for the LOP strategy and both the MRI_3mm and MRI_5mm strategy. It was tested if these differences were significantly different from 0 (*p* < 0.05) using the two-sided Wilcoxon signed-rank test in R.

## Results

### Patient data

Of the 17 patients that participated in this study, 14 received at least 3 weekly MRIs and were included in the analysis. In Table 1 characteristics of included patients are shown. In Table 2 an overview of relevant patient data is given. The average time per patient between the sagittal and transversal weekly MRI was between 8.0 min and 13.2 min (average of 10.0 min over all patients). For 5 patients, due to anatomical changes a CT for treatment planning was repeated during the course of the treatment and a new LOP was created. For 7 patients the pretreatment MRI was considered to be one of the weekly MRIs. The number of plans in the LOP ranged from 2 to 7.

**Table 1:** Patient characteristics

Patient number	Age	FIGO stage	Nodal stage (TNM)	Histopathology
1	49	IIB	N1	Small cell neuroendocrine carcinoma
2	50	IIB	N0	SCC
3	49	IB2	N1	SCC
4	60	IIB	N1	SCC
5	57	IB2	N1	SCC
6	38	IB2	N1	SCC
7	48	IB1	N0	Adenosquamous cell carcinoma
8	50	IIB	N0	SCC
9	54	IIB	N1	SCC
10	40	IIIB	N1	SCC
11	55	IIA2	N0	SCC
12	57	IIA2	N0	SCC
13	81	IIIA	N1	SCC
14	46	IB2	N1	SCC

*SCC = Squamous Cell Carcinoma*

**Table 2:** Overview of patient data. In the column with the plan selection, the plans in the LOP are labelled by the interpolation percentage, where 0 and 100 refer to the anatomy on the empty and full bladder scan, respectively

Patient number	Pretreatment MRI used for LOP?	Number of weekly MRIs	Average time (min) between weekly sagittal and transversal MRI	Number of plans in the LOP	Plan selection	Repeat CT?
1	Yes	4	12.0	6	40, 60, 100, 100	No
2	No	4	8.8	3	0, 0, 0, 0	Yes
3	No	5	10.2	2	100, 100, 0, 0, 100, 0	No
4	No	4	9.2	2	0, 0, 100	Yes
5	Yes	4	9.2	3	100, 100, 100, 100	No
6	No	4	9.6	4	0, 0, 0, 0, 0	No
7	No	4	13.2	7	17, 33, 0, 33, 33	No
8	No	3	9.5	2	0, 0, 100, 0	No
9	Yes	3	9.5	5	100, 75	Yes
10	Yes	3	8.0	2	100, 0	Yes
11	No	4	10.6	6	40, 40, 40, 20, 40	No
12	Yes	3	12.8	2	0, 100, 100	No
13	Yes	4	8.8	2	100, 0	Yes
14	Yes	5	9.3	7	0, 17, 0, 0, 0	No

### Estimated fraction dose including intrafraction motion

**Fig. 2:**
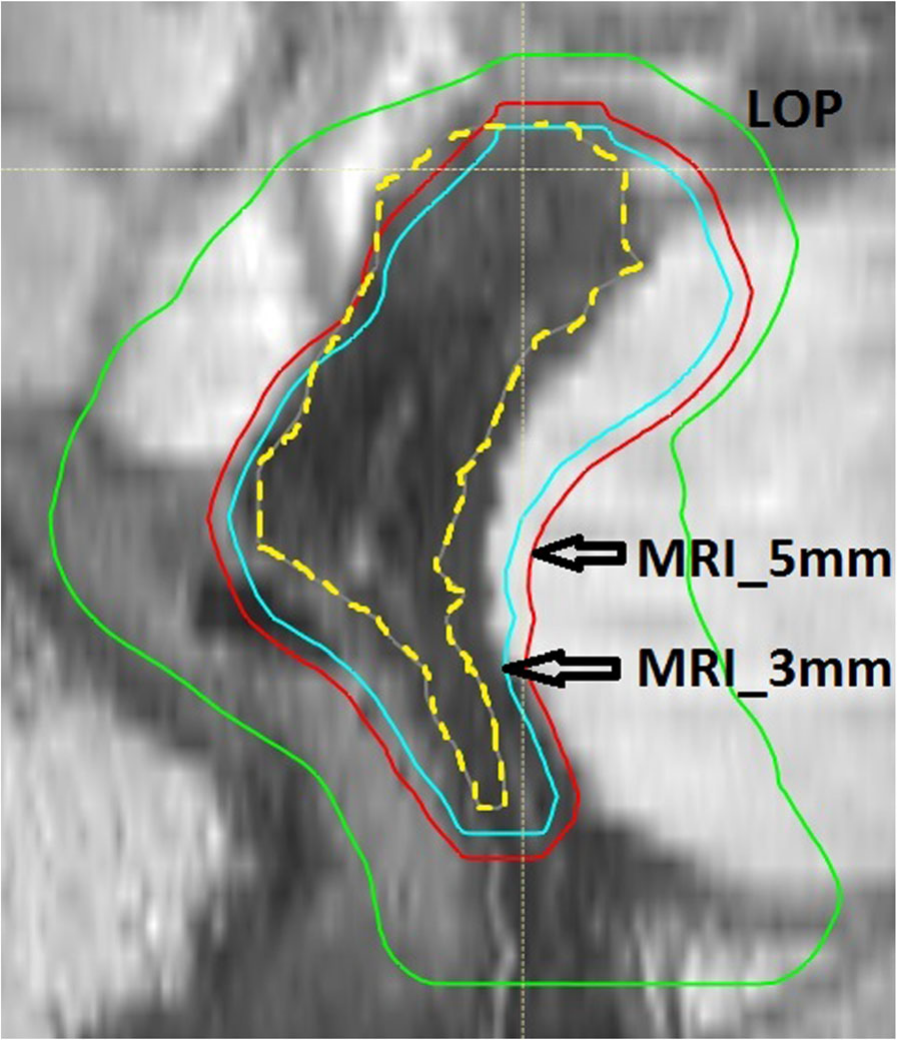
PTV of the selected plan in the LOP (green), and the PTVs of the MRI_5mm (red) and the MRI_3mm (blue) strategy overlaid on a transversal MRI. The low-risk CTV on the transversal MRI is indicated by the dashed yellow line. In this example, due to intrafraction bladder filling, the coverage of the low-risk CTV was insufficient for the MRI_3mm strategy (D_98%_ < 95%), while it was sufficient for the MRI_5mm and LOP strategy

In Tables 3, 4 and 5 the minimum D_98%_ is given per patient for the low-risk CTV (CTV_T_LR), the high-risk CTV (CTV_T_HR), and the lymph node regions CTV (CTV_LN_Pelvic). In case D_98%_ was less than 95%, the number of weekly MRIs for which D_98%_ ≥ 95% is also given, together with the total number of weekly MRIs. For the MRI_3mm strategy the D_98%_ for the low-risk CTV was less than 95% for at least one weekly MRI for 8 patients, while for the LOP and MRI_5mm strategy this was the case for 3 patients. In Fig. 2 an example is shown were the target coverage was insufficient for the MRI_3mm strategy due to intrafraction bladder filling, but sufficient for the MRI_5mm and LOP strategy.

**Table 3:** For each strategy the minimum D_98%_ for the low-risk CTV (CTV_T_LR) is given as a percentage of the prescribed dose. If the minimum value is less than 95%, also the number of weekly MRIs is given for which the D_98%_ ≥ 95%, as well as the total number of weekly MRIs

Low-risk CTV (CTV_T_LR)						
Patient number	D_98%_ LOP		D_98%_ MRI_3mm		D_98%_ MRI_5mm	
	Min. (%)	# ≥ 95%	Min. (%)	# ≥ 95%	Min. (%)	# ≥ 95%
1	93.1	3/4	93.8	3/4	96.7	
2	76.1	2/4	93.8	3/4	96.3	
3	96.6		96.2		96.8	
4	95.2		96.4		96.3	
5	95.8		94.7	3/4	96.5	
6	96.7		96.1		96.4	
7	89.4	4/5	81.0	2/5	81.6	4/5
8	96.5		96.3		96.9	
9	96.8		94.1	2/3	96.1	
10	95.6		95.9		96.0	
11	96.4		89.6	2/5	94.0	4/5
12	96.4		90.6	2/3	95.0	
13	96.6		93.1	3/4	95.0	3/4
14	96.6		95.2		96.2

**Table 4:** For each strategy the minimum D_98%_ for the high-risk CTV (CTV_T_HR) is given as a percentage of the prescribed dose. If the minimum value is less than 95%, also the number of weekly MRIs is given for which the D_98%_ ≥ 95%, as well as the total number of weekly MRIs

High-risk CTV (CTV_T_HR)						
Patient number	D_98%_ LOP		D_98%_ MRI_3mm		D_98%_ MRI_5mm	
	Min. (%)	# ≥ 95%	Min. (%)	# ≥ 95%	Min. (%)	# ≥ 95%
1	96.4		89.2	3/4	95.1	
2	82.5	2/4	89.2	3/4	96.5	
3	96.3		95.7		96.9	
4	97.0		96.6		96.4	
5	96.6		92.9	3/4	96.4	
6	97.0		96.4		96.5	
7	96.6		95.2		96.2	
8	96.7		96.3		96.9	
9	97.2		96.1		96.6	
10	95.2		96.1		95.5	
11	96.7		96.0		96.6	
12	96.4		95.4		96.1	
13	96.7		96.7		97.0	
14	96.9		95.6		96.0

**Table 5:** For each strategy the minimum D_98%_ for the lymph node regions CTV (CTV_LN_Pelvic) is given as a percentage of the prescribed dose. If the minimum value is less than 95%, also the number of weekly MRIs is given for which the D_98%_ ≥ 95%, as well as the total number of weekly MRIs

Lymph node regions CTV (CTV_LN_Pelvic)						
Patient number	D_98%_ LOP		D_98%_ MRI_3mm		D_98%_ MRI_5mm	
	Min. (%)	# ≥ 95%	Min. (%)	# ≥ 95%	Min. (%)	# ≥ 95%
1	96.1		96.0		96.5	
2	97.0		96.5		96.9	
3	96.8		96.6		96.6	
4	97.0		96.0		96.2	
5	96.9		93.6	3/4	94.1	3/4
6	97.3		96.4		96.5	
7	96.6		96.4		96.3	
8	96.8		96.0		96.2	
9	92.0	2/3	94.9	2/3	94.9	2/3
10	96.0		95.8		96.4	
11	95.1		88.2	3/5	88.3	3/5
12	96.6		94.6	2/3	94.1	2/3
13	90.3	2/4	92.5	2/4	92.6	2/4
14	96.9		97.2		96.5

For the MRI_5mm strategy the D_98%_ for the high-risk CTV was at least 95% for all weekly MRIs for all patients, while for the LOP and MRI_3mm strategy D_98%_ was less than 95% for at least one weekly MRI for 1 and 3 patients, respectively. For both MRI_3mm and MRI_5mm strategy D_98%_ was less than 95% for the lymph node regions CTV for at least one weekly MRI for 5 patients, while for the LOP strategy this was the case for 2 patients.

**Fig. 3:**
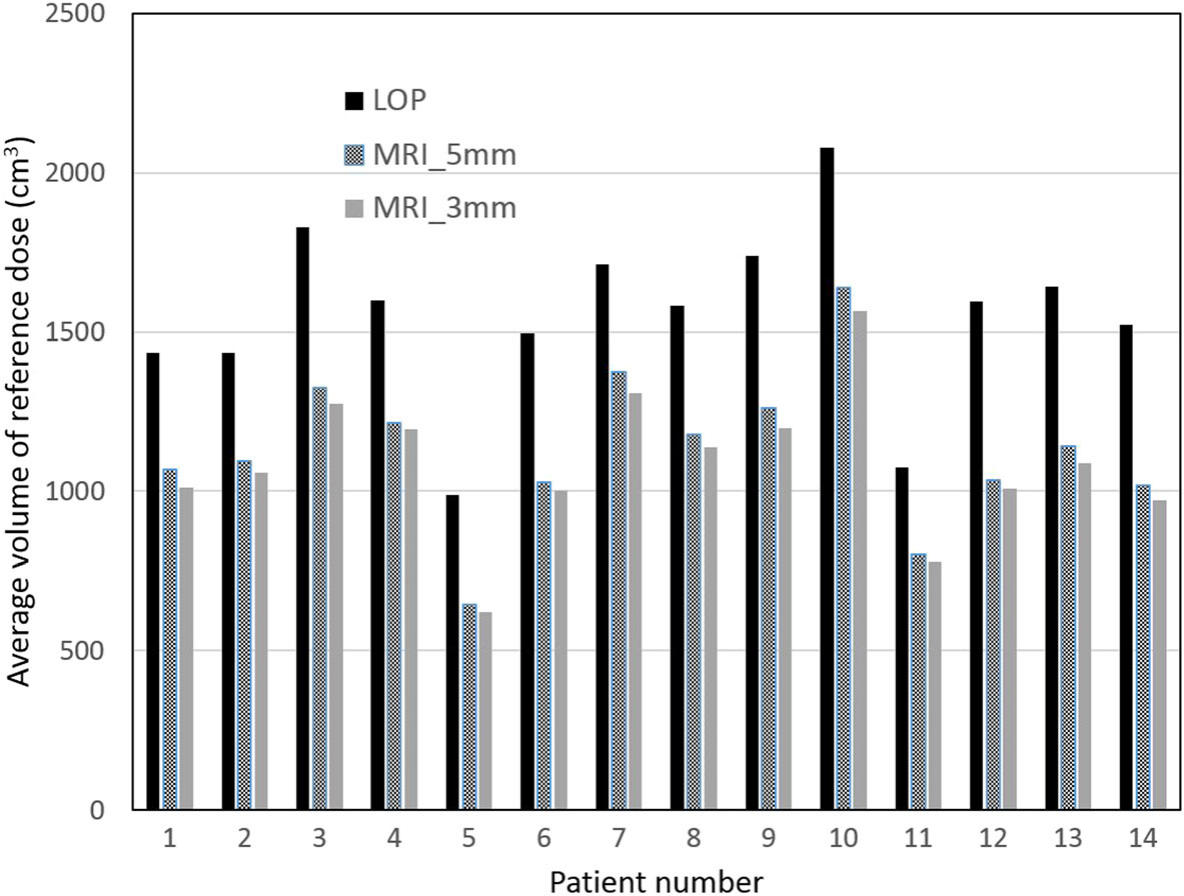
The average volume of the reference dose (95% of prescribed dose) for the different strategies

In Fig. 3 the volume of the reference dose (95% of prescribed dose) is given for each patient and each strategy. The average reduction of the volume of the reference dose as compared to the LOP strategy was 464 cm^3^ (range: 298 cm^3^ to 586 cm^3^) for the MRI_3mm strategy, and 422 cm^3^ (range: 273 cm^3^ to 559 cm^3^) for the MRI_5mm strategy.

**Fig. 4:**
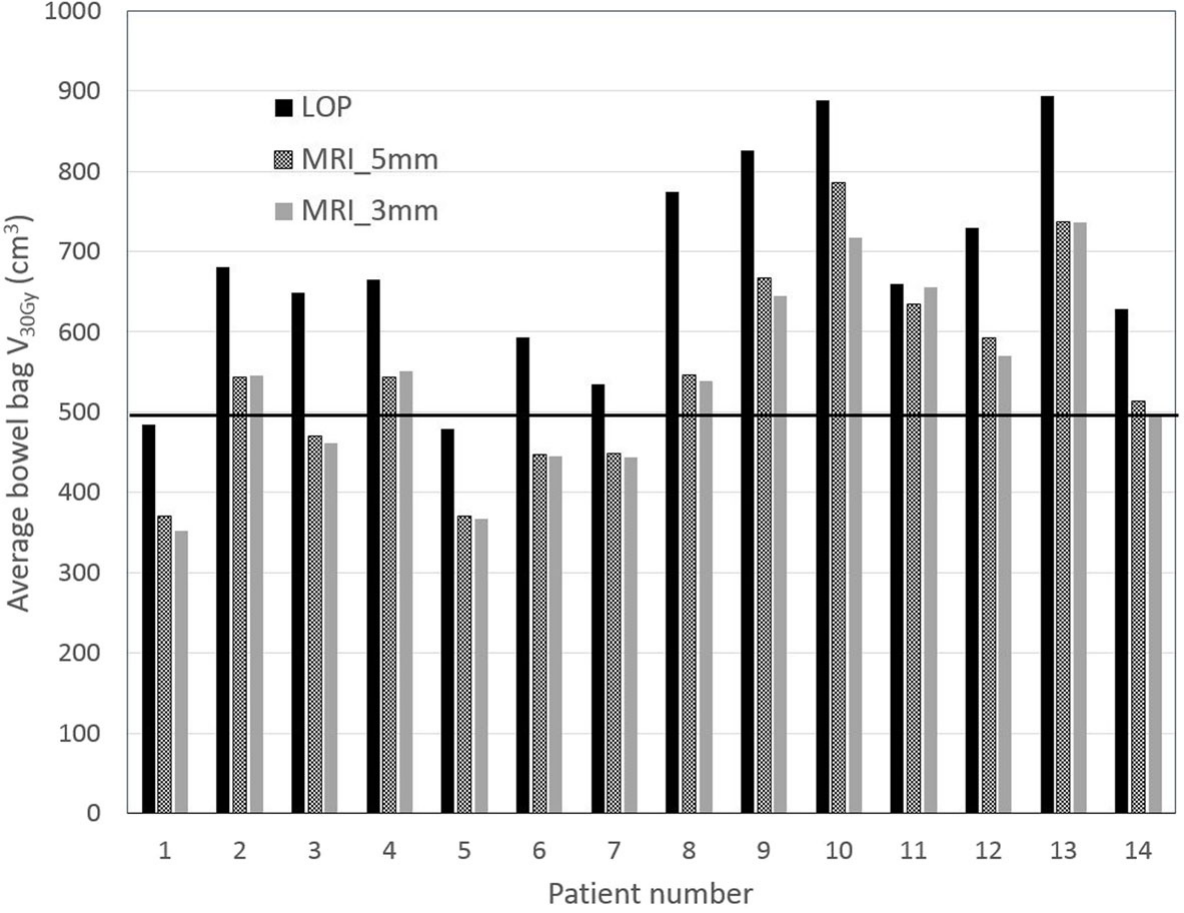
Average bowel bag V_30Gy_ of the estimated fraction dose for the different strategies. The dose constraint of Fiorino et al. is indicated by the horizontal line at 500 cm^3^

**Fig. 5:**
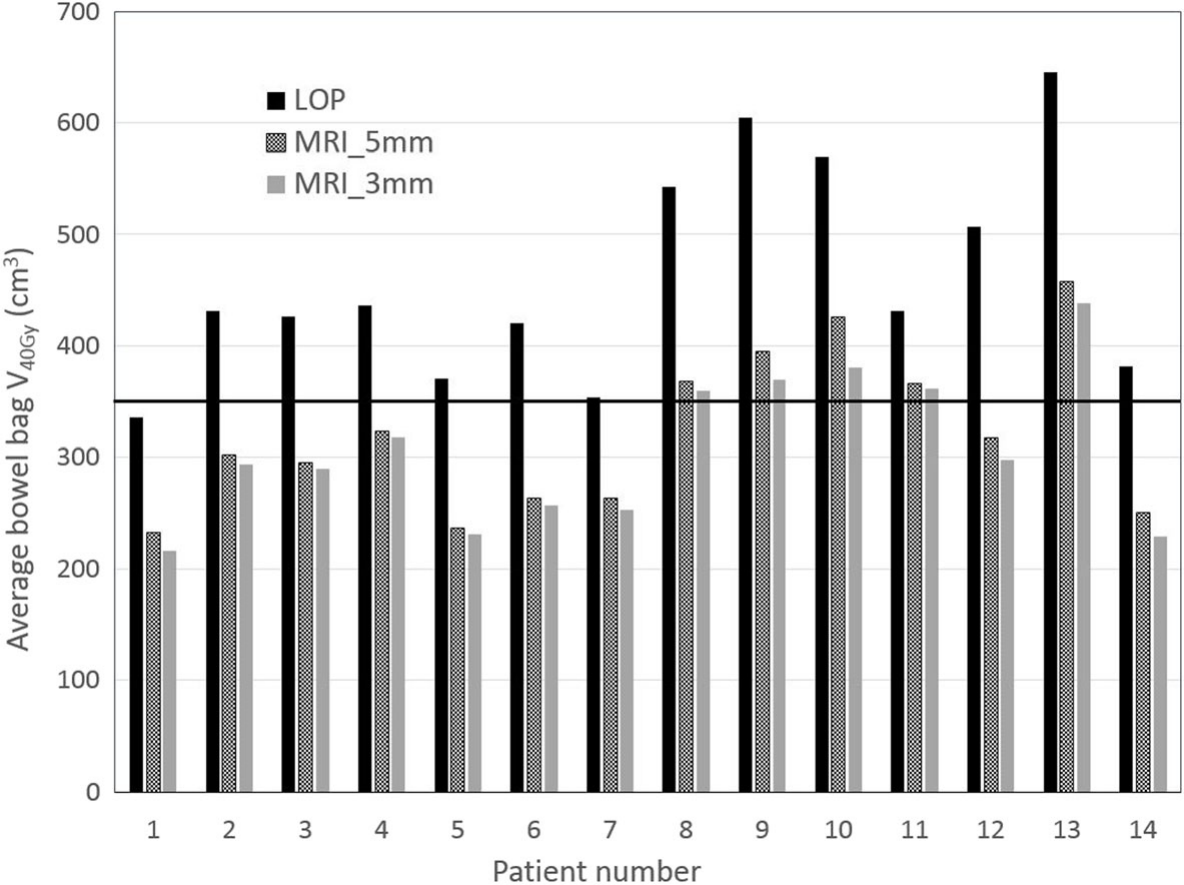
Average bowel bag V_40Gy_ of the estimated fraction dose for the different strategies. The dose constraint of Fiorino et al. is indicated by the horizontal line at 350 cm^3^

In Figs. 4 and 5 for each patient and each strategy the average bowel bag V_30Gy_ and V_40Gy_ is given, respectively, and also the constraint values suggested by Fiorino et al. [24] are indicated. For the LOP strategy the suggested constraint V_30Gy_ < 500 cm^3^ was violated for 12 of the 14 patients, while for the MRI_5mm and MRI_3mm this constraint was violated for 9 and 8 patients, respectively. The suggested constraint V_40Gy_ < 350 cm^3^ was violated for 13 patients for the LOP strategy and for 5 patients for both MRI_3mm and MRI_5mm strategy.

**Fig. 6:**
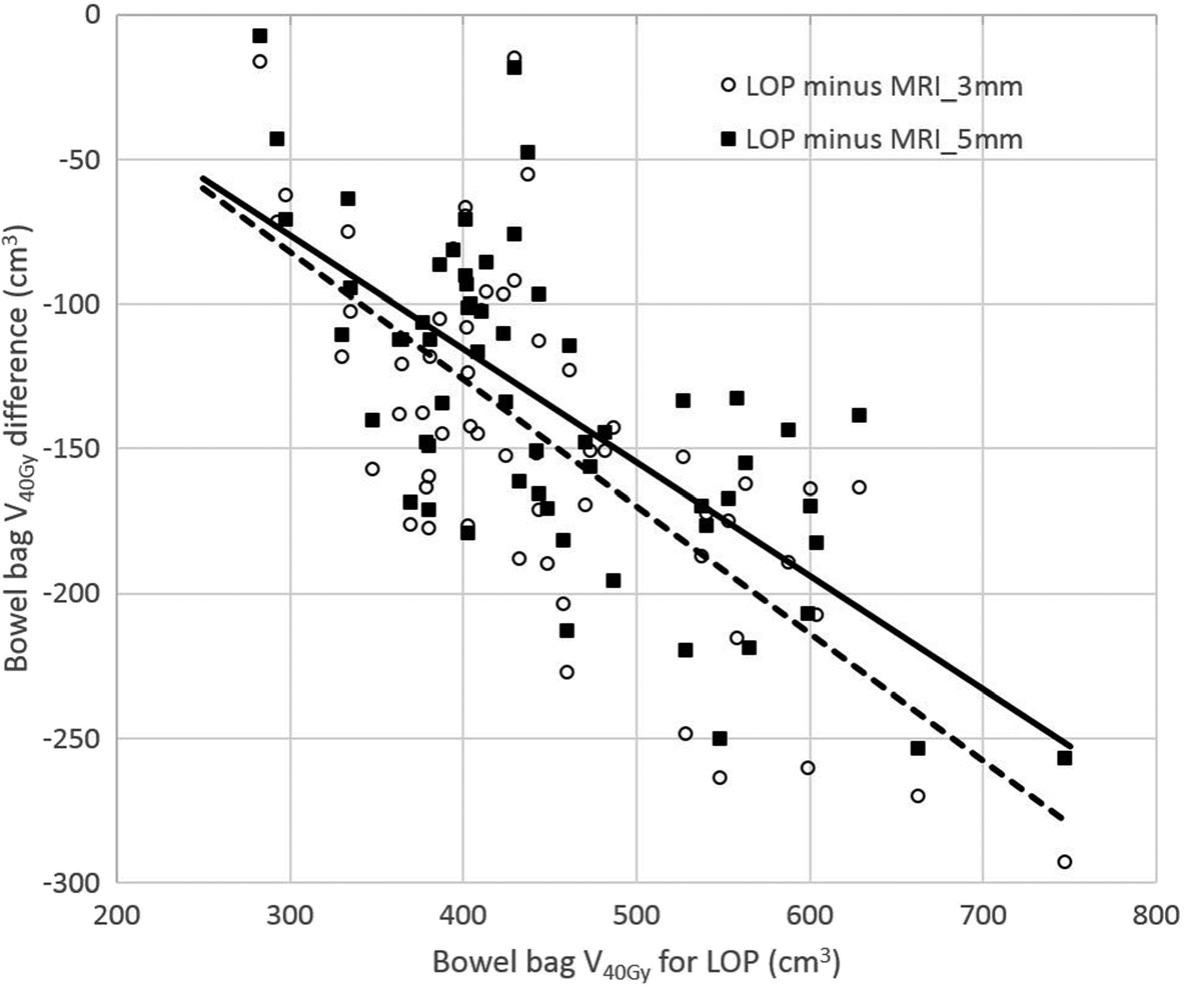
Bowel bag V_40Gy_ difference between LOP and both MRI_3mm and MRI_5mm strategy of the estimated fraction dose on the vertical axis and the bowel bag V_40Gy_ for the LOP strategy on the horizontal axis for all patients and all weekly MRIs. Also the fit from linear regression is shown (MRI_3mm strategy dashed line, MRI_5mm strategy full line)

In Fig. 6 the difference between bowel bag V_40Gy_ for the LOP and each of the MRI-guided strategy is shown as a function of the bowel bag V_40Gy_ for the LOP strategy, together with a linear fit. The linear coefficient of the linear regression model was − 0.44 and − 0.39 for the MRI_3mm and MRI_5mm strategy, respectively, and both were significantly different from 0 (*p* < 0.001), while the R^2^ statistic was 0.51 and 0.48, respectively.

In Additional file 1: Figure S1 boxplots are shown for the difference of the OAR DVH parameters between the LOP strategy and both MRI_3mm and MRI_5mm strategy. The average reduction of bladder V_40Gy_ was 24 and 17% (percentage points) for the MRI_3mm and MRI_5mm strategy, respectively, while the reduction in D_mean_ was 2.7 Gy and 1.8 Gy. For the rectum the average reduction of V_40Gy_ was 53 and 47% (percentage points), while for D_mean_ this was 14.0 Gy and 11.9 Gy, for the MRI_3mm and MRI_5mm strategy, respectively. The bowel bag V_40Gy_ was on average reduced by 148 cm^3^ and 135 cm^3^ for the MRI_3mm and MRI_5mm strategy, respectively, while for V_30Gy_ this was 136 cm^3^ and 129 cm^3^. For all evaluated OAR DVH parameters the differences between both MRI_3mm and MRI_5mm strategy and the LOP strategy were significantly different from 0 (*p* < 0.001).

### Evaluation of planned dose

In Additional file 1: Figure S2 boxplots are shown for PTV D_98%_, D_0.1%_ and CI for the planned dose. PTV D_98%_ was on average 95.3% for the LOP strategy, and 95.4% for both MRI_3mm and MRI_5mm strategy, where the differences between the strategies were not significant (*p* = 0.15). PTV D_0.1%_ was on average 103.8% for the LOP strategy, and 104.3% for both MRI_3mm and MRI_5mm strategy. The CI was on average 1.13, 1.19, and 1.18 for the LOP, MRI_3mm, and MRI_5mm strategy, respectively, while the target coverage requirement PTV V_95%_ > 98% was satisfied for all plans. Both PTV D_0.1%_ and CI were significantly different when comparing LOP and MRI_3mm, and LOP and MRI_5mm (both cases p < 0.001). PTV D_0.1%_ and CI were not significantly different when comparing MRI_3mm and MRI_5mm (*p* = 0.89 and *p* = 0.13, respectively).

## Discussion

In this study we found that for the MRI_3mm strategy for 8 out of 14 patients there was insufficient coverage of the low-risk CTV for at least one weekly MRI (Table 3), in exchange for only slightly more OAR sparing compared to the MRI_5mm strategy. However, the high-risk CTV seems less affected by intrafractional motion, as underdosage for the MRI_3mm strategy occurred only in 3 out of 14 patient for one weekly MRI, compared to no underdosage for the MRI_5mm strategy (Table 4). Additionally, the MRI_5mm strategy has a target coverage comparable to the LOP strategy and therefore has the best trade-off between coverage and dose to organs at risk in our group of patients.

Kerkhof et al. studied the benefit of MRI guidance for the OAR as compared to using a single IMRT plan created pretreatment [17]. While their study focussed on the OAR, our study also considers the target coverage, and includes the effect of intrafraction motion. Furthermore, as a major addition to the work of Kerkhof et al. our study compares a MRI-guided strategy with a LOP, which is the current state of the art.

In our study the average time interval between the sagittal and transversal weekly MRI was 10.0 min. In our clinic the time interval that starts after the acquisition of the Cone Beam CT (CBCT) and ends after the VMAT fraction delivery of the selected plan in the LOP is less than 10 min, so the time interval in this study is possibly too large for the LOP strategy. For the MRI-guided strategy this time interval might not be feasible yet, although the time interval between MRI acquisition and availability of a reoptimized treatment plan was on average only 12 min in a study by Bohoudi et al. [25] .

Kerkhof et al. studied intrafraction motion of the cervix-uterus over a time interval of 4, 9, and 16 min, using MRI [26]. After 9 min, which is comparable to the time interval in our study, they found that for 50% of all patients and all MRIs, the cervix-uterus motion was less than 3.1 mm, while for 90% this was less than 7.8 mm. Our study showed comparable results, since we found insufficient CTV_T_LR coverage for at least one weekly MRI for 8 out of 14 patients for the MRI_3mm strategy, and 3 out of 14 for the MRI_5mm strategy (Table 3). In the study of Heijkoop et al. intrafraction motion of cervix-uterus was studied using pre- and post-fraction CBCTs, where the time interval was 20.8 min on average, which is representative of the delivery of an Intensity-Modulated Radiation Therapy fraction [16]. They found considerable intrafraction motion, up to 10 mm and larger, but these results could not be compared to our study, because the time interval in their study was much larger than in our study.

Heijkoop et al. found that the intrafraction random and systematic setup translation error along the different patient axes was between 0.4 mm and 1.4 mm for an average time interval of 20.8 min [16]. In our study the average time interval was 10.0 min and these errors are expected to be less than 1 mm. In this study intrafraction setup translations were not taken into account by removing the translations between the sagittal and transversal weekly MRIs. For the LOP strategy the time interval between CBCT acquisition and fraction delivery is smaller than the time interval between the sagittal and transversal MRI. Therefore, including translations between sagittal and transversal MRI would overestimate the intrafraction setup translations. On the other hand, for the MRI-guided strategy the time interval between the planning MRI and the fraction delivery is probably larger. It is likely that, as part of an MRI-guided strategy, an MRI would be acquired immediately prior to the fraction delivery to verify setup and correct for possible setup translations that have occurred after acquisition of the planning MRI. This also justifies removing the translations between the sagittal and transversal MRI.

In contrast to intrafraction setup translations, intrafraction setup rotations were taken into account in this study, which affected the coverage of the lymph node regions CTV (Table 5). For both MRI_3mm and MRI_5mm strategy a PTV margin of 3 mm was applied to the lymph node regions CTV, which resulted in an underdosage for 6 out of 14 patients for at least one weekly MRI, while the 5 mm PTV margin used for the LOP strategy was insufficient for 2 patients.

In this study the planned dose distribution was used to calculate the DVH parameters for the delineations on the weekly transversal MRI. This way, the dosimetric effect of anatomical changes and setup rotations between the planning CT and the weekly MRIs was not taken into account. However, since this affects the results for the LOP and MRI-guided strategy in an identical way, the effect on the results of the comparison of these strategies is expected to be small. Also, for 5 patients in this study a repeat CT was acquired because of anatomical changes and the dose distribution on the repeat CT was used for the evaluation of the weekly MRIs that were taken after the repeat CT. This means that for these patients anatomical changes were taken into account to some extent. Sun et al. studied the effect of body contour changes on the position of isodose lines for prostate VMAT [27]. They found that the 95% isodose line shifts by less than 1 mm in case of a body contour change of 1 cm in all directions but posterior, which shows that the effect of these kind of anatomical changes is quite small.

A considerable reduction of small bowel toxicity might be achieved with the MRI-guided strategy compared to the LOP strategy, as we found that the dose constraint V_40Gy_ < 350 cm^3^, suggested by Fiorino et al. [24], was satisfied for 9 out of 14 patients for the MRI-guided strategy, compared to 1 patient for the LOP strategy. In our study the V_40Gy_ reduction for the MRI-guided strategy is most beneficial when the V_40Gy_ for the LOP strategy is relatively large (Fig. 6). This confirms former research of our group wherein proton therapy and reduced CTV strategies show most benefit for patients with a V_45Gy_ of the small bowel > 200–275 cm^3^. In this group of patients, a decrease of > 10% in NTCP for grade ≥ 2 acute small bowel toxicity is expected [28]. As acute small bowel toxicity is also a risk factor for late small bowel toxicity, reduction of acute small bowel toxicity potentially has impact in lifetime quality of life for these women [12]. Roeske et al. [29] developed a Normal Tissue Complication Probability (NTCP) model for acute gastrointestinal toxicity, for which V_45Gy_ was the relevant DVH parameter. In our study the prescribed dose was 45 Gy and the treatment plans were homogeneously planned. As a consequence, for all plans created in this study the V_45Gy_ was very small, as can be seen in Additional file 1: Figure S3. Since this would result in unrealistically low NTCP values, the NTCP model of Roeske et al. was not used in this study.

For this study contour-based deformable registration was used to generate interpolations of the low-risk CTV. However, deformable registration was not used to accumulate dose, and the dosimetric analysis was purely based on fraction dose. Voxel-to-voxel correspondence with the present deformable registration methods is probably not accurate enough for dose accumulation [30]. Therefore, although for all strategies there was under dosage of the different CTVs, no hard conclusions can be drawn from this about the target coverage for the whole treatment. However, for the MRI-guided strategy with a 5 mm PTV margin for the low-risk CTV the coverage of the high-risk CTV was sufficient for all weekly MRIs, and the coverage of the low-risk CTV was insufficient only for three weekly MRIs for three different patients (Tables 3 and 4). Therefore, it is likely that for the MRI-guided strategy a PTV margin larger than 5 mm is not needed. For the bowel bag we compared average V_30Gy_ and V_40Gy_ with the dose constraints suggested by Fiorino et al. [24]. We think that taking the average V_30Gy_ and V_40Gy_ over the weekly MRIs results in good estimates of the V_30Gy_ and V_40Gy_ for the whole treatment, and is preferred over using deformable registration to accumulate dose.

The CI and D_0.1%_ for the planned dose were significantly higher for the MRI-guided strategy than for the LOP strategy. The higher CI is mostly explained by the fact that the volumes of the MRI-guided PTVs were much smaller than the volumes of the LOP PTVs. Also, because for the LOP plans an ITV margin was applied, the PTV shape was in general less complex than the PTV shape of the MRI-guided plans. As a consequence, the 95% isodose was less conformal to the PTV for the MRI-guided strategy. For a more complex PTV shape, it is in general also more difficult to achieve a homogeneous dose in the PTV, which might also explain why the D_0.1%_ was slightly higher for the MRI-guided plans.

In order to achieve a bladder filling on the weekly MRIs that is representative of a treatment fraction, the patients were asked to follow the same drinking instructions prior to the acquisition of a weekly MRI as prior to each treatment fraction. It was not investigated if the patients observed the drinking instructions prior to the weekly MRIs. Because of logistical reasons, weekly MRIs were acquired either before or after a treatment fraction. It was not investigated if bladder fillings on weekly MRIs acquired before and after the treatment fraction were different. Multiple patients had a (nearly) empty bladder on all weekly MRIs (see Plan Selection column in Table 2), which does not represent the clinical situation, where the patients were encouraged to have a comfortably filled bladder during treatment delivery in order to reduce dose to the bowel bag. This might affect the applicability of the results of this study in the clinic.

Patient 2 had an empty bladder on the weekly MRIs, while the bladder filling was larger on the empty CT scan that was used for the clinically used LOP. As a consequence, on all weekly MRIs the uterus was outside the PTVs of the LOP. However, during the actual treatment there was sufficient target coverage using the LOP because of better bladder filling. Since the weekly MRIs did not represent the actual clinical situation, the first weekly MRI was used to create the LOP, instead of the clinically used empty bladder CT. This was the only patient for which another scan was used to generate the LOP than in the clinic.

During delineation, consistency of the delineated target volume on the weekly MRIs was carefully considered. Remaining delineation uncertainties were a bias in favor of the MRI-guided strategy in this study, since it is likely that the delineations on sagittal and transversal MRI acquired during the same scanning session were, because of almost identical anatomy, more consistent than for MRIs of different weeks. Furthermore, there might be a systematic difference between delineations on CT, which were used for the LOP, and delineations on MRI.

Pathologic lymph nodes were not taken into account in this study. At our institute pathologic lymph nodes receive an integrated boost to 55.0 Gy or 57.5 Gy, which is in accordance with the EMBRACE II study protocol [20]. For the LOP strategy the PTV margin for the lymph node region was 5 mm, while for the MRI-guided strategy 3 mm was used. Therefore, if pathologic lymph nodes had been taken into account, it is expected that, because of the smaller PTV margin for the MRI-guided strategy, there would relatively be more sparing of the OAR for the MRI-guided strategy. However, due to intrafraction motion, the PTV margin for the lymph node region that was used for the MRI-guided strategy was not large enough to achieve sufficient coverage for all weekly MRIs in this study.

## Conclusions

A considerable sparing of the OAR can be achieved when an online MRI-guided strategy is used for EBRT of cervical cancer, as compared to a LOP strategy. If a new treatment plan can be generated and delivered within 10 min, an online MRI-guided strategy with a 5 mm PTV margin for the low-risk CTV is sufficient to account for intrafraction anatomical changes.

## Additional file

Additional file 1: **Table S1.** Wish list for the automatic generation of the plans in the LOP. **Table S2.** Wish list for the automatic generation of the plans for the MRI strategy. **Figure S1.** DVH parameters of the OAR for the estimated fraction dose. **Figure S2.** PTV D_98%_, PTV D_0.1%_ and CI for the planned dose for the sets of all LOP, MRI_3mm and MRI 5_mm plans. **Figure S3.** For each strategy a box plot is shown for the bowel bag V_45Gy_ of the estimated fraction dose for all weekly MRIs and all patients. (DOCX 207 kb)

## Data Availability

The datasets generated and/or analyzed during the current study are not publicly available since the participants did not consent in sharing the data with third parties.

## Abbreviations

CI: Conformity Index
CTV: Clinical Target Volume
DVH: Dose Volume Histogram
EBRT: External Beam Radiation Therapy
FOV: Field Of View
ITV: Internal Target Volume
LOP: Library of Plans
MRI: Magnetic Resonance Imaging
MRI_3mm: MRI-guided strategy with 3 mm PTV margin for CTV low-risk
MRI_5mm: MRI-guided strategy with 5 mm PTV margin for CTV low-risk
NTCP: Normal Tissue Complication Probability
OAR: Organ at Risk
PTV: Planning Target Volume
VMAT: Volumetric-Modulated Arc Therapy
