# Supplementary material for: Dosimetric comparison of library of plans and online MRI-guided radiotherapy of cervical cancer in the presence of intrafraction anatomical changes
Source: Radiat Oncol. 2019 Jul 12;14:126. doi: 10.1186/s13014-019-1322-0 (PMC6624982; doi:10.1186/s13014-019-1322-0)
Supplement: Supplementary file 1 — Table S1. Wish list for the automatic generation of the plans in the LOP. Table S2. Wish list for the automatic generation of the plans for the MRI strategy. Figure S1. DVH parameters of the OAR for the estimated fraction dose. Figure S2. PTV D98%, PTV D0.1% and CI for the planned dose for the sets of all LOP, MRI_3mm and MRI 5_mm plans. Figure S3. For each strategy a box plot is shown for the bowel bag V45Gy of the estimated fraction dose for all weekly MRIs and all patients. (DOCX 207 kb) [file 13014_2019_1322_MOESM1_ESM.docx]

Supplemental Material:

Dosimetric comparison of library of plans and online MRI-guided radiotherapy of cervical cancer in the presence of intrafraction anatomical changes

J. Visser, P. de Boer, K.F. Crama, Z. van Kesteren, C.R.N. Rasch, L.J.A. Stalpers, A. Bel

# Tables

Table S1: Wish list for the automatic generation of the plans in the LOP.

| **Structure name** | **Clinical goal** | **Priority** |
| --- | --- | --- |
| PTV | D_98%_ ≥ 42.75 Gy (95% of 45 Gy) | 1 |
| External | D_1cm_^3^ ≤ 47.25 Gy (105% of 45 Gy) | 1 |
| Bowel bag | Reduce average dose as much as possible | 2 |
| External | Dose Fall-Off | 3 |
| Bladder | D_0.1%_ ≤ 47.25 Gy | 4 |
| Bowel bag | D_0.1%_ ≤ 47.25 Gy | 4 |
| Rectum | D_0.1%_ ≤ 47.25 Gy | 4 |
| Bladder | Reduce average dose as much as possible | 5 |
| Rectum | Reduce average dose as much as possible | 5 |

Table S2: Wish list for the automatic generation of the plans for the MRI strategy.

| **Structure name** | **Clinical goal** | **Priority** |
| --- | --- | --- |
| PTV | D_98%_ ≥ 42.75 Gy (95% of 45 Gy) | 1 |
| External | D_1cm_^3^ ≤ 47.25 Gy (105% of 45 Gy) | 1 |
| Bowel bag + bladder | Reduce average dose as much as possible | 2 |
| External | Dose Fall-Off | 3 |
| Bowel bag + bladder | D_0.1%_ ≤ 47.25 Gy | 4 |
| Rectum | D_0.1%_ ≤ 47.25 Gy | 4 |
| Rectum | Reduce average dose as much as possible | 5 |

# Figures


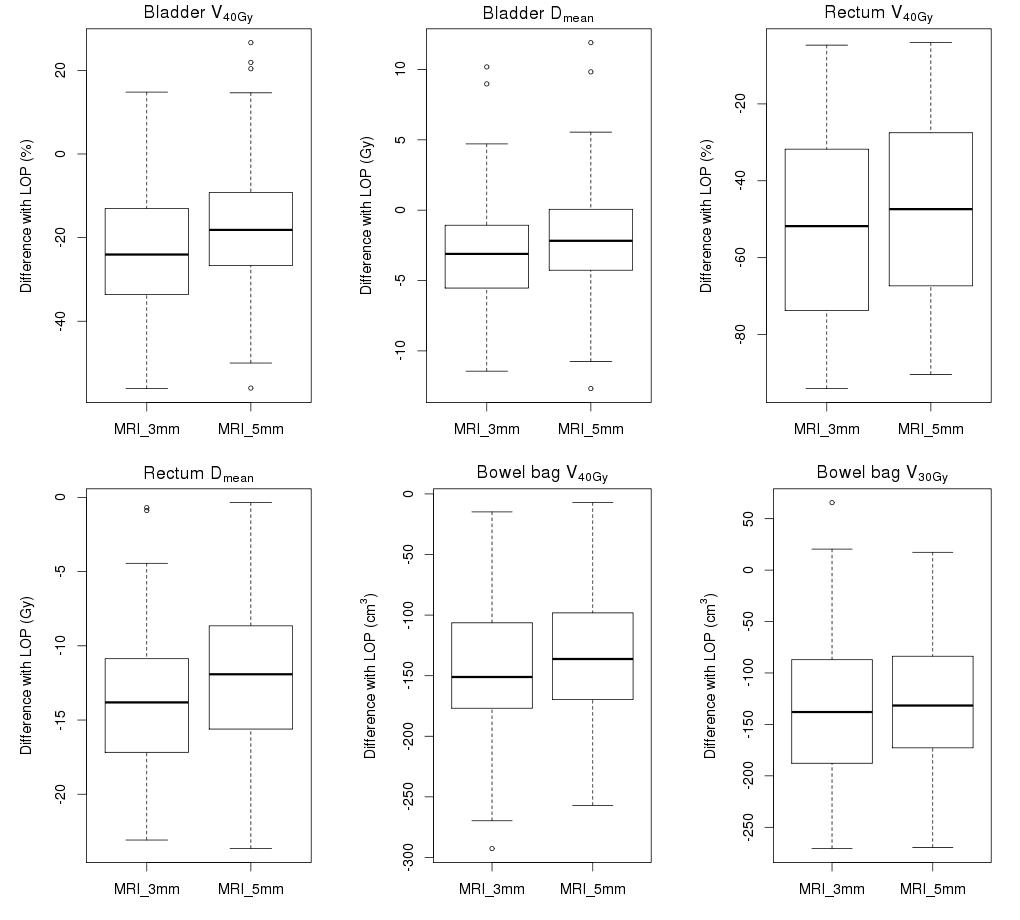


Figure S1: DVH parameters of the OAR for the estimated fraction dose. The difference is shown between the MRI_3mm and LOP strategy, and between the MRI_5mm and LOP strategy. Negative values indicate more sparing for the MRI strategies. Percentage differences are to be interpreted as percentage point differences. Boxes: median value, and lower and higher quartiles; whiskers: lowest and highest data point within 1.5 times the inter-quartile range from the quartiles; dots: outliers.


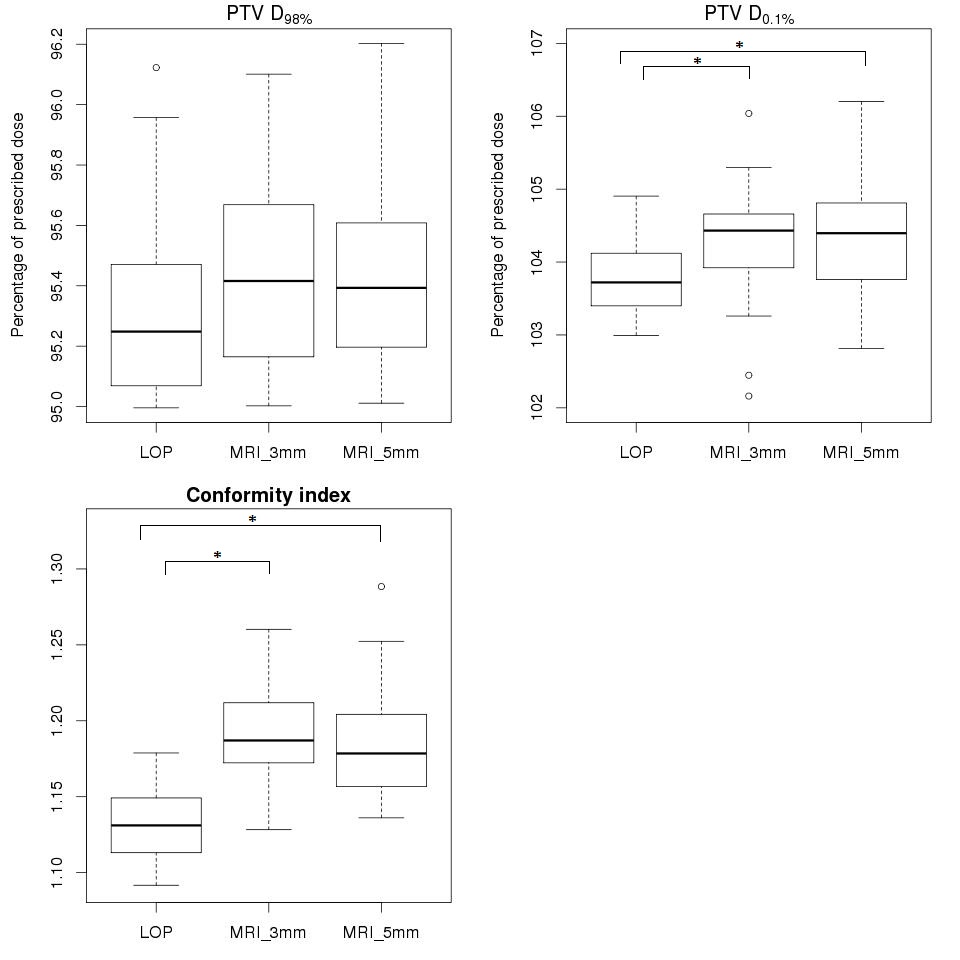


Figure S2: PTV D_98%_, PTV D_0.1%_ and CI for the planned dose for the sets of all LOP, MRI_3mm and MRI 5_mm plans. Significant differences between groups are indicated by an asterisk.


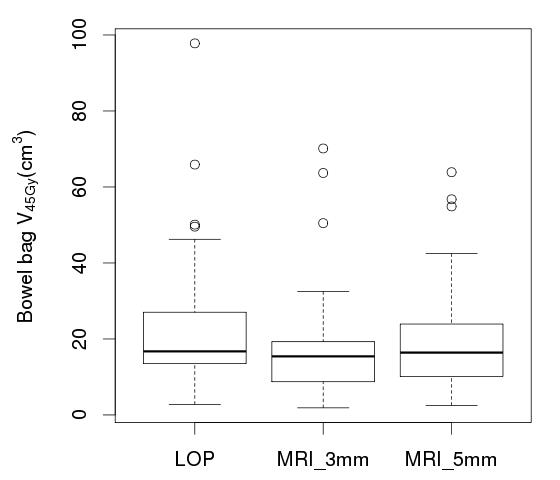


Figure S3: For each strategy a box plot is shown for the bowel bag V_45Gy_ of the estimated fraction dose for all weekly MRIs and all patients.
